# Imiquimod-Loaded Nanosystem for Treatment Human Papillomavirus-Induced Lesions

**DOI:** 10.3390/pharmaceutics16070864

**Published:** 2024-06-27

**Authors:** Izamara Maocha, Beatriz Rosado, Jéssica Lopes-Nunes, Melanie Lopes, Joana Rolo, Bruno Pires, Eugénia Gallardo, Ana Palmeira-de-Oliveira, José Martinez-de-Oliveira, Rita Palmeira de Oliveira, Rui Medeiros, Carla Cruz

**Affiliations:** 1 CICS-UBI—Health Sciences Research Centre, University of Beira Interior, 6201-506 Covilhã, Portugal; izamaocha@gmail.com (I.M.); beatriz.rosado@ubi.pt (B.R.); jessicalonu@hotmail.com (J.L.-N.); melanie.lopes@ubi.pt (M.L.); joanarolo@fcsaude.ubi.pt (J.R.); bruno.mg.pires@gmail.com (B.P.); egallardo@fcsaude.ubi.pt (E.G.); apo@fcsaude.ubi.pt (A.P.-d.-O.); jmo@fcsaude.ubi.pt (J.M.-d.-O.); rpo@fcsaude.ubi.pt (R.P.d.O.); 2 Labfit–Health Products Research and Development Lda, UBIMedical, 6200-284 Covilhã, Portugal; 3 Faculty of Health Sciences, University of Beira Interior, 6200-506 Covilhã, Portugal; 4 Molecular Oncology and Viral Pathology Group, Research Center of IPO Porto (CI-IPOP)/RISE@CI-IPOP (Health Research Network), Portuguese Oncology Institute of Porto (IPO Porto)/Porto Comprehensive Cancer Center (Porto.CCC) Raquel Seruca, 4200-072 Porto, Portugal; ruimedei@ipoporto.min-saude.pt; 5 Faculty of Medicine, University of Porto (FMUP), 4200-319 Porto, Portugal; 6 Departamento de Química, Universidade da Beira Interior, 6201-001 Covilhã, Portugal

**Keywords:** human papillomavirus, liposomes, DNA aptamer, imiquimod, vaginal formulation, essential oils

## Abstract

Human papillomavirus (HPV)-associated cervical cancer is the most common cancer among women worldwide. The treatment options are strongly related to increased infertility in women. Imiquimod (IQ) is an imidazoquinoline, which has proven antiviral effects against persistent HPV infection by activating immune cells via Toll-like receptors 7/8 when formulated in carriers, like nanogels, for topical use. An effective alternative to conventional therapies is the nanoparticle drug delivery system. We studied lipidic nanoparticles with IQ (Lipo IQ) and functionalized them with a DNA aptamer, AT11 (Lipo IQ AT11), to improve the selectivity for cervical cancer cells combined with the efficacy of essential oils. The formulations showed that the physicochemical properties are adequate for vaginal drug delivery and have antimicrobial activity at higher concentrations (with MIC_50_ starting from 0.625%). The final formulations exhibited cytotoxicity in cancer cells, enhanced by essential oils without affecting healthy cells, resulting in less than 10% cell viability in HeLa cells and over 60% in NHDF cells. Essential oils potentiate Lipo IQ’s effectiveness, while AT11 increases the selectivity for cervical cancer cells. As suggested by the results of the permeation assay, the formulations were internalized by the cancer cells. Overall, the obtained results suggested that the synergistic effect of the essential oils and the nanosystem potentiate the cytotoxic effect of Lipo IQ and that Lipo IQ AT11 promotes selectivity towards cancer cells.

## 1. Introduction

The most common human papillomavirus (HPV)-associated cancer among women worldwide is cervical cancer [1,2]. Vaccination does not treat or cure precancerous lesions in women already infected with the virus and is, therefore, ineffective for a large part of the population [3]. The treatment options for cervical dysplasia are strongly related to increased infertility in women [3]. As well as being invasive, these methods have limited efficiency and undesirable side effects [2]. A global strategy approved in August 2020 by the World Health Organization (WHO) outlines three key goals to achieve by 2030, aiming to reduce the incidence of cervical cancer to less than 4 cases per 100,000 women, thereby eliminating cervical cancer as a public health problem [4,5]. One of these goals is the necessary and appropriate treatment for women who are identified with precancerous lesions or cervical cancer [4]. Thus, improved treatment options for women already infected with the virus are still needed. 

The use of essential oils combined with other therapies has been widely explored given its anticancer and antimicrobial properties [6,7]. Due to its complex composition, such as terpenes, terpenoids, aliphatics, and aromatics, they have shown great interest in the pharmaceutical industry [7,8]. Several preclinical studies point to the anticancer potential of *Thymus vulgaris* and *Oreganum vulgare* through different mechanisms of action, including suppressing cell growth, inducing apoptosis, producing intracellular reactive oxygen species, and depolarizing the membrane potential [6,9], which are mainly associated to the activity of thymol and carvacrol, the major components of these essential oils and its derivatives.

IQ is an imidazoquinoline and can activate the immune cells through Toll-like receptors 7 and 8 (TLR7/8) to produce proinflammatory cytokines/interferons [10] and it is an effective drug in treating lesions associated with HPV infection [11]. IQ is being used as a cream in a topical immune response modifier with indirect antiviral and antitumor properties [12]. It is prescribed for HPV-associated genital warts, superficial basal cell carcinoma, and actinic keratosis [10,12]. Studies showed that it is a safe and effective treatment for the usual type of vulvar intraepithelial neoplasia (VIN), which is pathophysiologically comparable to cervical intraepithelial neoplasia (CIN) [13]. Furthermore, IQ seems to also be effective in primary CIN lesions [14]. Also, it has been formulated in nanoparticles, showing that nanosystems containing IQ present a more cytotoxic effect against cervical cancer cells and were able to reduce the production of inflammatory cytokines by up to 25% in comparison to free IQ [15]. In this way, nanoparticle-based targeted and drug delivery systems have increasing therapeutic efficacy and reduced drug side effects [16]. There have been significant upgrades in optimizing delivery systems to overcome poor solubility and cellular uptake [17]. Liposomes fall into the category of nanomedicine and have been used to deliver DNA, RNA, and antigens [17]. Their interesting properties such as biocompatibility, amphiphilic drug loading, and targeting effect are some of the advantages of using them as a safe drug delivery system [18,19]. Previously, Akhtar et al. carried out a study in which they confirmed the potential of delivering drugs into HPV-infected cervical cancer cells using an optimized liposome drug carrier, and they observed a higher ratio of drug toxicity uptake in the cells [20].

Aptamers are small RNA or DNA oligonucleotide sequences that exhibit high affinity and specificity against different targets [21]. Most notably, AS1411 has gained attention in anticancer research due to its ability to bind nucleolin (NCL), a protein found in the nucleus of proliferating cells and on the cell surface of cancer cells [22,23]. Phan et al. described that a simple base substitution (G to T) can form a new aptamer termed AT11, which adopted a single major G4 conformation, while still exhibiting similar antiproliferative activity as AS1411 [24]. AT11 has shown to be promising in the development of selectivity cancer-targeted systems for the delivery of anticancer drugs [22]. 

Therefore, it was hypothesized that AT11 liposome (Lipo AT11) could serve as a promising carrier for IQ in cervical cancer cells when combined with essential oils, aiming to treat intraepithelial lesions caused by HPV. 

**Figure 1:**
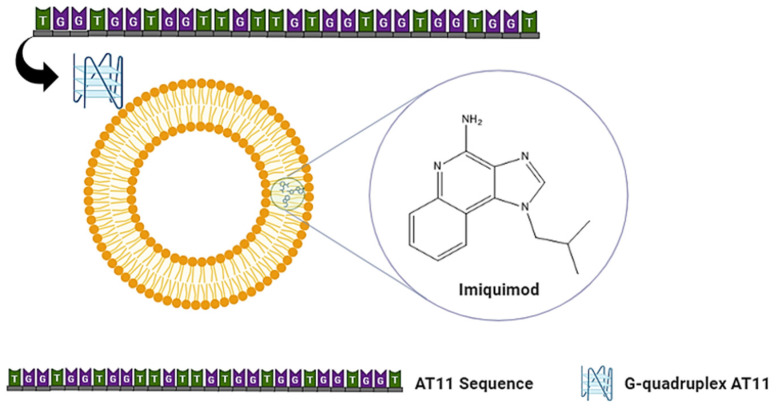
Schematic representation displaying the structure of Lipo IQ AT11.

In this study, the synthesis and characterization of the Lipo IQ AT11 was described (Figure 1), and its potential to selectively deliver IQ to the cancer cells was evaluated. The resulting formulations, incorporating the nanosystem, were also characterized by different physicochemical methods, and their vaginal penetration capacity was evaluated. Additionally, their anticancer and microbiological activities were determined, and the cellular uptake was analyzed using confocal microscopy.

## 2. Materials and Methods

### 2.1. Materials

Cholesterol (CAS 57-88-5) was purchased from Sigma-Aldrich (St. Louis, MO, USA), and phosphatidylcholine (PHOSPHOLIPON^®^ 90G) was acquired from Lipoid Kosmetik™ (Steinhausen, Switzerland). The lipidic solutions were prepared with 99.5% ethanol with a concentration of 12.5 mg/mL for cholesterol and 130 mg/mL for phosphatidylcholine. The AT11 aptamer (5′-TGG-TGG-TGG-TTG-TTG-TGG-TGG-TGG-TGG-T-Cholesteryl-TEG-3′) and Cy5-labeled AT11 aptamer (5′-Cy5-AT11-Cholesteryl-TEG-3′) were purchased from Eurogentec (Seren, Belgium). The stock solution was prepared using Milli-Q water and stored at −20 °C until use. The concentration of the oligonucleotide was determined by measuring the absorbance at 260 nm using a UV–Vis spectrophotometer (Thermo Scientific™ Evolution 220, MA, USA) and the molar extinction coefficient (ε) provided by the manufacturer. IQ (1-Isobutyl-1H-imidazo[4,5-c] quinoline-4-amine; CAS: 99011-02-6; purity > 98%) was purchased from Tokyo Chemical Industry (Zwijndrecht, Belgium), and a 2 mM stock solution was prepared with a palmitic acid and ethanol solution prepared with a 0.92 mg/mL concentration to improve the drug solubilization. Potassium sorbate (CAS 24634-61-5|105119) was purchased from Merck (Darmstadt, Germany), hydroxypropylmethyl cellulose (Methocel K100) from DOW Chemical Company, Midland, MI, USA, propylene glycol (CAS 57-55-6) was acquired from LaborSpirit, Lda, (Santo Antão do Tojal, Portugal), and polysorbate 80 (Tween^®^ 80, CAS: 9005-65-6) was obtained from Thermo Fisher Scientific^TM^ (Waltham, MA, USA). The essential oil of *Thymus vulgaris* and *Oreganum vulgare* was acquired from Blossom Essence^®^ (Covilhã, Portugal) and Elegante (Viseu, Portugal), respectively. Normal human dermal fibroblasts (NHDF, PCS-201-012™), human cervical cancer cells (HeLa, CCL-2™), SiHa cells (HTB-35™), and CaSki cells (CRM-CRL-1550™) were obtained from ATCC (USA). RPMI (Roswell Park Memorial Institute) 1640 medium and DMEM (Dulbecco’s Modified Eagle Medium) were purchased from Corning Life Sciences (Glendale, AZ, USA). MEM (Minimal Essential Medium) was purchased from Gibco, Thermo Scientific™ (MA, USA), and supplemented with 1% of MEM Non-Essential Amino Acid Solution (100×) from Sigma Aldrich (St. Louis, MO, USA). 3-[4,5-dimethylthiazol-2-yl]-2,5 diphenyl tetrazolium bromide (MTT) (CAS 298-93-1) and dimethyl sulfoxide (DMSO) (CAS 67-68-5) were acquired from Thermo Fisher Scientific™ (Waltham, MA, USA).

### 2.2. Production and Characterization of the Liposomes

#### 2.2.1. Production of the Liposomes

The liposomes were produced using the ethanol injection method described by Batzri and Korn [25], using two different lipids. The lipidic solution was prepared by mixing 375 µL of cholesterol (from a stock solution of 12.5 mg/mL, corresponding to 4.69 mg), 375 µL of phosphatidylcholine (from a stock solution of 130 mg/mL, corresponding to 48.75 mg), and 750 µL of IQ (from a stock solution at 2 mM). Then, 1 mL of the lipidic solution was injected through a syringe with an optical fiber coupled, using a NE-300 Just Infusion Syringe Pump (New Era Pump Systems, Farmingdale, NY, USA), in 9 mL of Milli-Q water. It took 30 min and 18 s for a total of 1 mL of the injected lipid solution. Finally, a final liposomal solution with IQ at 100 µM was obtained. The solution was stored at room temperature and wrapped in aluminum foil to protect it from the light.

The liposomes were functionalized with AT11-TEG-Cholesteryl by incubating them with the oligonucleotide stock solution (1 mg of liposomes to 1 nmol of oligonucleotide) for 20 min at room temperature [26,27]. The functionalization was confirmed by performing an acrylamide gel with SYBR™Gold (Thermo Fisher Scientific™, Waltham, MA, USA) as the nucleic acid stain. Then, they were stored at 4 °C for further use.

#### 2.2.2. Structural Characterization of the Liposomes

The previously produced nanoparticles were characterized by Dynamic Light Scattering (DLS) using Zetasizer Nano ZS equipment (Malvern Instruments, Malvern, UK). Briefly, the average size and the polydispersity index (PDI) were determined by placing 1 mL of liposomes into a quartz glass cuvette. Data were collected at a constant temperature of 25 ± 1 °C. The data presented correspond to the average of the measurements of three samples.

#### 2.2.3. Liposomes’ Drug Loading and Release Characterization

Two different assays were performed to assess the pharmacokinetics of the nanoparticles. Drug loading was performed to determine the quantity of IQ integrated into the lipidic membrane and was obtained with centrifugal concentrators (Sartorius, Göttingen, Germany; MWCO of 2 kDa). A standard calibration curve was established with different concentrations of IQ to further determine the amount of drug incorporated in the liposomes. The absorbance of the standard samples was measured by spectrophotometry at a wavelength of 318 nm with a UV–Vis spectrophotometer (Thermo Scientific™Evolution 220, Waltham, MA, USA) [28]. For the drug release assay, a standard calibration curve was established with different concentrations of IQ to further determine the exact amount of the drug released by the liposomes. The fluorescence of the standard samples was measured with a Horiba FluoroMax^®^ 4 Fluorometer, Osaka, Japan. A total of 100 μL of Lipo IQ were inserted in a Slide-a-LyzerTM (Thermo Fisher Scientific™, Waltham, MA, USA; MWCO of 3.5 kDa), and the dialysis device was further inserted in a 1.5 mL Eppendorf previously filled with 1 mL of a phosphate-buffered saline solution (PBS). The samples were under agitation at room temperature using a PTR-35 Multi-Rotator (Thermo Fisher Scientific™, Waltham, MA, USA), and 100 μL of the buffer was collected and replaced at different time points (0 min, 5 min, 15 min, 30 min, 1 h, 2 h, 3 h, 4 h, 5 h, 6 h, 12 h, 24 h, and 72 h). The fluorescence of the collected samples (λ_ex_ = 318 nm and λ_em_ = 333 nm) was measured to further calculate the percentage of the drug released.

### 2.3. Preparation of the Vaginal Formulations and the Vaginal Fluid Simulant

The excipients used in the preparation of the gel formulations are Generally Recognized as Safe (GRAS, FDA) pharmaceutical-grade materials (European Pharmacopoeia 9.0). Potassium sorbate was used as a preservative element, hydroxypropylmethylcellulose (HPMC k100) as a polymer, and propylene glycol as a permeation promotor/humectant. The composition was based on the Universal Placebo, a gel previously shown to be safe for vaginal application [29]. A total of 0.1% (*w*/*w*) of the potassium sorbate was dissolved in water in constant agitation using a helical stirrer (Heidolph RZR 2041, Heidolph Instruments GmnH & Co., Schwabach, Germany). After it was dissolved, 1.5% (*w*/*w*) HPMC was added, as well as the remaining water. In the end, 8% (*w*/*w*) of propylene glycol was inserted into the mixture also under agitation. The pH of the gel was adjusted to 5.5 with NaOH 1 M, and the resulting mixture was centrifuged at 3000 rpm for 5 min at 4 °C. The TEO and OEO formulations were prepared following the same procedure by dispersing 0.25% (*w*/*v*) or 1% (*w*/*v*) of OEO or TEO mixed with Tween 80 1% (*v*/*v*) for emulsification purposes [30]. These formulations were prepared with just the essential oils; TEO (base formulation with essential oil of *Thymus vulgaris)* and OEO (base formulation with essential oil of *Oregano vulgare)* were used as the controls.

To prepare the formulation with Lipo IQ AT11, a concentration of 1% was previously established so that the concentration of IQ in the formulation was 1 µM. For this, the amount corresponding to 1% of the total volume was incorporated into the formulation with TEO (TEO IQ AT11) or OEO (OEO IQ AT11) and gently mixed.

The vaginal fluid simulant (VFS) was prepared as described by Owen and Katz in 1999 [31]. To prepare the modified vaginal fluid simulant (mVFS), 1.5% (*w*/*w*) of porcine gastric mucin-type II was added to the VSF previously prepared.

### 2.4. Physiological Characterization of the Formulations

The gel formulations were characterized by different parameters: pH and buffering capacity, viscosity, osmolality, and bioadhesion. For the viscosity and osmolality assays, measurements were made directly on the formulations, as well as physiological dilutions, where 1.5 g of each formulation was diluted in 225 µL of VFS at the physiological vaginal temperature (37 °C), considering the expected volume of commercialized product in the applicator (5 g) and the estimated volume of VFS present in the vagina at any moment (0.75 mL).

#### 2.4.1. pH and Buffering Capacity

The pH of the formulations was measured at room temperature using an electrode of pH (Thermo Scientific Orion Star A211 pH meter, Thermofisher, Waltham, MA, USA), and then the buffering capacity assay was performed. For that, 1 g of formulation was diluted in 10 mL of 0.9% NaCl under agitation. This buffering capacity is used as a control. Since the pH of this sample has a pH > 5, the reverse buffering capacity was assessed by adding increments of 20 µL of hydrochloric acid (HCl) 1 M until reaching a pH < 3.

The same procedure was performed using VFS as the dispersion medium and 1 g of formulation to clarify the buffering capacity in the vaginal environment. Starting at a pH of around 4.5, increments of 20 µL of sodium hydroxide (NaOH) 1 M were added until reaching a pH > 9.

#### 2.4.2. Viscosity

Viscosity was assessed using a conical plate rheometer (Brookfield DV-3T, Brookfield, Middleboro, MA, USA). The measurements were performed on the direct formulations after dilution in VFS at the physiological vaginal temperature (37 °C) in triplicates for 1 min, and were then left to rest for 1 min between measurements. The cones used were CPA-52Z with a cone angle of 3° and a radius of 1.2 cm, respectively (Brookfield, Middleboro, MA, USA), and 500 µL of the formulation was placed on the plate. To obtain an acceptable torque (10–100%), the rotation speeds were between 5 and 250 RPM, and the samples were left to equilibrate for 1 min between measurements [32].

#### 2.4.3. Osmolality

Osmolality was determined using a freezing point osmometer (Osmomat 3000, Gonotec, Berlin, Germany) and 50 µL aliquots of direct and diluted VSF formulations. Standardization was performed using three standards: distilled water (point zero), NaCl 300 mOsm/kg, and NaCl 850 mOsm/kg, commercially available from the equipment manufacturer.

#### 2.4.4. Bioadhesion

To evaluate the formulation adhesiveness in the porcine vaginal tissue, a texturometer (TAXT Plus, Stable Micro Systems, Godalming, UK) was used, which evaluated the tensile strength of the interfacial layer formed between the formulation and the vaginal epithelium. To determine this strength, a cylindrical probe with a diameter of 10 mm (P10) was used. The measurements were made in an oven at 37 ± 1 °C to mimic the physiologic temperature. The epithelium samples were fixed in the base of the equipment with the help of the mucoadhesive device and then were hydrated with 50 µL of mVFS, assuming that mucin is the protein responsible for bioadhesion. A double-sided adhesive tape was used to attach a small piece of the cellulose acetate membrane to the probe, where it was directly weighed 30 mg of the formulation. The cellulose membrane without formulation was used as a control. The software was used in adhesive mode connected to the texturometer according to the conditions previously described [32]. The negative graphic area and the detachment force were recorded, representative of the adhesion work (N.mm) that is essential to separate the two surfaces.

### 2.5. Evaluation of Formulations in the Culture Medium

The formulations were diluted to concentrations of 20%, 15%, 10%, 5%, 1%, 0.4%, 0.2%, and 0.1% (*w*/*v*) in 1 mL of DMEM supplemented with 10% fetal bovine serum and 1% antibiotic. To ensure the solubilization of the formulations, 0.5% DMSO was included. The formulations were then left in contact with the culture medium for 48 h.

### 2.6. In Vitro and Ex Vivo Cell Assays

#### 2.6.1. Cell Culture

HeLa, SiHa, CaSki (cervical cancer cells), and NHDF (normal human dermal fibroblasts) were used in this work. HeLa cells were cultivated in DMEM, NHDF and CaSki cells were cultivated in RPMI-1640 medium, and SiHa were cultivated in MEM (supplemented with 1% Non-Essential Amino Acid Solution). All culture media were supplemented with 10% fetal bovine serum (FBS) and 1% penicillium/streptomycin antibiotic. These cultures were then incubated at 37 °C in a humidified atmosphere containing 5% of CO_2_.

#### 2.6.2. Cell Viability Assay

Cell viability was assessed through the colorimetric MTT assay, measuring the percentage of viable cells after the incubation with the formulations. The formulations were diluted to concentrations of 15%, 10%, and 5% (*w*/*v*) in 1 mL of the respective medium, each containing 0.5% DMSO to ensure the solubilization of the formulations.

Cells were seeded on 96-well plates at a density of 10 × 10^4^ cells/mL with the corresponding culture medium and incubated for 24 h. Following the incubation, the medium was removed, and 100 µL of the formulations were added for an additional 24 h. Control wells included untreated cells and wells with medium containing 0.5% DMSO. Afterward, the medium was removed, and 100 µL of an MTT solution (1 mg/mL) was added. HeLa cells were incubated for 45 min, CaSki and SiHa cells for 2 h, and NHDF cells for 15 min and 1 h. Subsequently, the MTT was removed, and the formazan crystals were dissolved in DMSO. Absorbance was measured using a plate reader (Bio-rad xMark spectrophotometer from Bio-Rad, Hercules, CA, USA) at 570 nm.

#### 2.6.3. Confocal Fluorescence Microscopy

For the fluorescence confocal microscopy assay, the same cell lines were used, seeded at 5 × 10^4^ cells/mL in 8 wells (IBIDI, Gräfelfing, Germany) in 200 μL of medium, and grown at 37 °C under a 95% air and 5% CO_2_ humidified atmosphere. After 24 h of incubation, the cell lines were washed three times with PBS, and nuclei were stained with a Hoechst 33342 1 μM nuclear probe for 15 min. Then, the cells were treated with imiquimod-loaded nanoparticles AT11 labeled with Cy5 and with the nanoparticle incorporated in the essential oils’ formulations for 24 h. The probe was washed and rinsed again with PBS three times, and images were collected using a Zeiss AxioObserver LSM 710 microscope with 405 and 633 nm laser excitation for Hoechst 33342 and AT11 labeled with Cy5 visualization, respectively.

#### 2.6.4. Permeation of the Formulations in Vaginal Tissue (Ex Vivo)

Vaginal epithelial tissue was acquired from fresh porcine tissue from a slaughterhouse and was cut using a roughly 300 μm thick dermatome. The permeation assay was performed using NaviCyte horizontal 9 mm circular Ussing chambers with an exposed tissue surface area of 0.64 cm^2^ (Harvard Apparatus, Holliston, MA, USA). The receptor chamber was filled with 1.8 mL of VFS. To balance the membrane, 200 μL of VFS was added into the donor chambers. After 20 min, VFS was replaced with 300 mg of the formulations, except the control. A total of 100 μL of each formulation were collected from the receptor chambers at the same times of the release assay: 0 min, 5 min, 15 min, 30 min, 1 h, 2 h, 3 h, 4 h, 5 h, 6 h, 12 h, 24 h, 48 h, and 72 h. The epithelial tissue and the samples at the end of 72 h were also collected for further analysis by the high-performance liquid chromatography (HPLC) method.

### 2.7. HPLC Analysis

In this study, the quantification of IQ compound concentrations was carried out using an Agilent Technologies 1290 HPLC system equipped with a 1260 fluorescence detector (FLD) (Soquimica, Lisbon, Portugal). Chromatographic separation of analytes was achieved using a YMCTriart PFP (5 µm, 4.6 i.d. × 150 mm) analytical column connected to a guard holder (4 × 10 mm) and a Triart PFP (5 µm, 3 × 10 mm) precolumn sourced from Solítica (Lisbon, Portugal). The mobile phase, comprising methanol with 0.1% trifluoroacetic acid in water (9:1; *v*/*v*), was delivered isocratically at a flow rate of 0.5 mL/min. The entire chromatographic run lasted 20 min, with an injection volume of 100 µL. The column and autosampler temperatures were maintained at 25 and 4 °C, respectively. The detection of analytes occurred at 318 nm (excitation) and 333 nm (emission). The retention time was 10.7 min.

### 2.8. Microbiology Assay: Minimal Inhibitory Concentration

The minimal inhibitory concentration (MIC) is the lowest concentration of a compound that inhibits the growth of the microorganisms and was determined based on the method of microdilutions following the rules of CLSI (Clinical & Laboratory Standards Institute). Both visual MIC and MIC_50_ were assessed. Visual MIC refers to the last blue well in the column of the desired condition, and MIC_50_ was calculated with both 570 nm and 620 nm absorbances and represents the necessary concentration to inhibit at least 50% of microbial activity. The microorganisms used were the bacteria *Staphylococcus aureus* (ATCC 6538), the yeast *Candida albicans* (ATCC 10231), and a filamentous fungus *Aspergillus brasiliensis* (ATCC 16404). The culture mediums used were Trypticase Soy Agar (TSA) and Mueller Hinton Broth (MHB) for bacteria, Sabourad dextrose agar (SDA) and RPMI-1640 for the yeast, and potato dextrose agar (PDA) for the fungus, as well as RPMI-1640.

#### 2.8.1. *Staphylococcus aureus*

The strain was inoculated in a Tryptone Soy Agar (TSA) plate and was incubated at 37 °C. The suspension was prepared the same way as the *Candida albicans* strain, the only difference being that 200 μL of inoculum and 19.8 mL of Mueller Hinton Broth (MHB) medium were used, resulting in a 1:100 dilution of the suspension (set to 0.5 McFarland, which corresponds to 1 × 10^8^ CFU/mL). The 96-well plate was prepared as previously described. A total of 100 μL of the bacterial suspension was added in all the wells that contained formulation dilutions, as well as in the positive control. Resazurin solution was also added to the wells, and the absorbance at 570 nm and 620 nm was measured after 4 h of incubation.

#### 2.8.2. *Candida albicans*

About 24 h before the test, the yeast strain was inoculated in a Sabouraud dextrose agar (SDA) plate and incubated at 37 °C. The inoculum was prepared by suspending isolated colonies in a NaCl (0.85%) solution, and the OD was set to 0.5 McFarland (1 × 10^6^ CFU/mL), measured with a Grant Bio DEN-1 densitometer (Grant Instruments, Cambridge, UK). Afterward, a 1:20 dilution was prepared using 1 mL of inoculum and 19 mL of the RPMI medium. The 96-well plate was prepared as previously described. A total of 100 μL of the yeast suspension was added in all the wells that contained formulation dilutions, as well as in the positive control. Resazurin solution was also added to the wells, and the absorbance at 570 nm and 620 nm was measured after 4 h of incubation.

#### 2.8.3. *Aspergillus brasiliensis*

Firstly, the fungus was inoculated in a potato dextrose agar (PDA) plate for 4 to 7 days before the test and maintained in the incubator at 25 °C (NuAIRE, Plymouth, MN, USA). After incubation, the colonies were covered with 1 mL sterile NaCl (0.85%) solution, as well as one drop of Tween 20, and the suspension was mixed and transferred to an Eppendorf. From this mixture, 100 μL was removed and mixed with 900 μL of RPMI-1640 medium to measure the optical density at a 600 nm wavelength. The suspension was set to an OD_600_ value between 0.09 and 0.11 by applying the C_i_ × V_i_ = C_f_ × V_f_ formula and a 1:50 dilution was performed, obtaining a final value of 0.4–5 × 10^4^ CFU/mL. The formulations were diluted in the RPMI medium at twice the concentration as the concentration pretended in the first row of wells, that is, a 20% concentration was prepared so that the first well had a 10% concentration of formulation compared with the medium. In a 96-well plate, 200 μL of the sample was placed in the first row of the plate, as well as 100 μL of the medium in the remaining wells. A ratio of 1:2 dilutions was performed by pipetting 100 μL of the previous well into the next, successively, up to the F row, and the remaining volume was discarded. G and H rows were used for positive and negative controls that only contained the medium plus the microorganism and the medium, respectively. The plate was incubated at 37 °C (BINDER, Neckarsulm, Germany). After 24 h, the wells were resuspended, and 30 μL of the resazurin solution (0.01% in sterile water, ThermoFisher Scientific, MA, USA) was added, followed by an incubation time of around 4 h at 37 °C. Absorbance at 570 nm and 620 nm was read in a Bio-Rad xMark spectrophotometer plate reader (Bio-Rad Laboratories, Hercules, CA, USA).

### 2.9. Statistical Analysis

All the data collected that comprised more than one sample were presented as either a mean ± SEM or SD, according to the experiment. Statistical significance was evaluated using one-way ANOVA and two-way ANOVA, and *p*-values ≤ 0.05 were considered significant. All data were analyzed using GraphPad Prism version 8.0 (GraphPad Software, La Jolla, CA, USA).

## 3. Results and Discussion

### 3.1. Production and Characterization of Liposomes

Liposomes with appropriate sizes can influence the absorbability of target tissues [18]. Phospholipids have several advantages as a safe drug delivery system, including biodegradability, biocompatibility, and drug-carrying capacity into HPV-infected cervical cancer cells, when compared to other nanoparticles [18,20]. Moreover, they can maintain therapeutic levels of the drug over time [20], which could further improve treatment results.

As described in the methods section, the liposomes were produced using the ethanol injection method with two different lipids and the active compound IQ (Figure S1) at a final concentration of 100 µM. Then, they were characterized using Dynamic Light Scattering (DLS) to assess the variation in hydrodynamic size, polydispersity index (PDI), and zeta potential on the day they were prepared, and after 30 days stored at different temperatures, four samples were measured: blank liposomes (BLs), Lipo IQ, blank-AT11 liposomes (BL AT11), and Lipo IQ AT11. These measurements were made on the day of synthesis and one month after for stability purposes. The results of the average size and polydispersity index (PDI) are presented in Table 1, showing that the liposomes have an appropriate size between 50 and 200 nm, with a PDI equal to or below 0.3, according to the literature [33]. The liposome particle size distribution diagram is shown in Figure S2. When compared with other nanoparticle-based delivery systems for imiquimod, namely, polymeric nanocapsules, the liposomes showed better results in terms of hydrodynamic mean diameters and the polydispersity index, thus being more suitable for cell internalization [34,35]. As reported, IQ is associated with the liposome membrane due to its hydrophobic structure, and it is explained by the smaller size of Lipo IQ compared with BL. When functionalized with the AT11 aptamer, both liposomes increased in size. These results were expected since AT11 is conjugated into the surface of the pre-formed liposomes via hydrophobic interactions of cholesterol with the bilayer of liposome membranes [26]. In addition, the hydrophilic properties of the aptamers and their size force them to the surface of the liposomes. The surface charge was also measured for all samples. The zeta potential value of the BL and Lipo IQ was −4.67 mV and −9.26 mV, respectively. After aptamer conjugation, the zeta potential was −11.37 mV for BL AT11 and −12.70 mV for Lipo IQ AT11. As a result of the negative nature of the aptamer, the zeta potential of both types of liposomes was lower when attached to AT11 than when not. The BL with a negative surface charge might be due to the presence of ethanol that serves as a source of negative charge [36].

After 30 days, a decrease in size can be observed when comparing the AT11-functionalized liposomes with the ones without the aptamer. This can be due to both AT11 liposomes being stored at 4 °C and the others at room temperature.

#### 3.1.1. Functionalization of Liposomes with AT11

To confirm the binding of the AT11 aptamer to the surface of the liposomes, a fixed concentration of Lipo IQ was incubated with an increasing concentration of the AT11 aptamer (1 or 2 nmol) and then analyzed by acrylamide gel electrophoresis. Free AT11 (lane 1) was used as a control for comparison. Comparing the trapping of the conjugates to the unbound aptamer is shown in Figure S3. As a negatively charged single-strand DNA, AT11 can freely transfer itself through the gel from the negative to the positive electrode, as can be seen in lane 2, showing the characteristic bands of the aptamer. The large molecular weight of the conjugate prevents AT11 from migration and stays trapped in the well, as can be seen in lanes 3 and 4, resulting in a decrease in the intensity of the AT11-characteristic bands [26]. This means that the post-insertion method of the aptamer was successful. The observed streak of bands in lane 4 (2 nmol of the aptamer to 1 mg of liposomes) suggests an excess of free aptamers, indicating that the concentration of the aptamer at 1 nmol to 1 mg of liposomes seems to be the appropriate ratio.

These results are in line with the previous studies [26], as the absence of an aptamer band means that it was efficiently conjugated, and the band’s intensities increase with the concentrations of the conjugated aptamer.

#### 3.1.2. Liposome IQ Loading and Release

**Figure 2:**
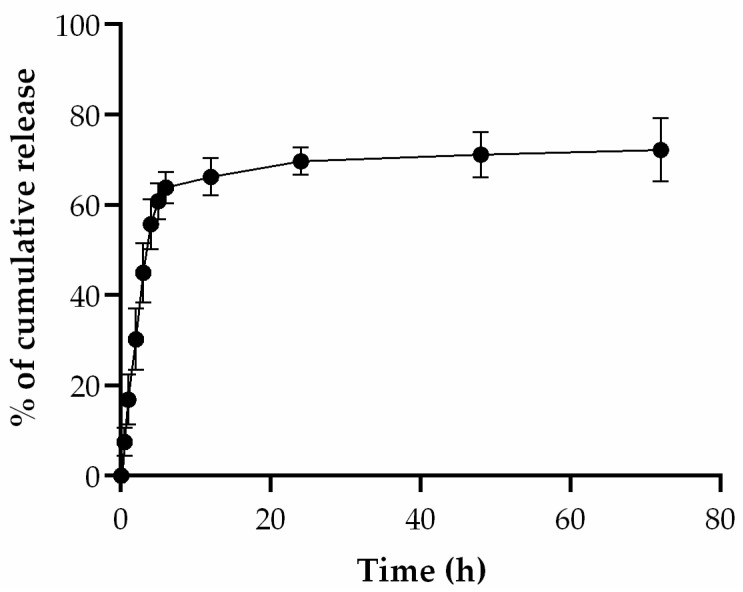
Cumulative release of IQ from liposomes in PBS at pH 7.4 (*n* = 3).

For the drug loading assay, a standard calibration curve with different concentrations of IQ was established by measuring the samples at a 318 nm wavelength, as shown in Figure 2. From these data, the percentage of non-encapsulated IQ was 20.8%. The drug release assay was performed to evaluate the release profile of IQ from the liposomes in PBS over 72 h. As seen in Figure 2, IQ was almost fully released from the liposomes during the first 24 h, suggesting that the drug can be successfully delivered to cancer cells after this time. Since the purpose is a topical formulation to be applied in the vaginal cavity, these results are favorable.

### 3.2. Physiological Characterization of the Formulations

#### 3.2.1. pH and Buffering Capacity

**Figure 3:**
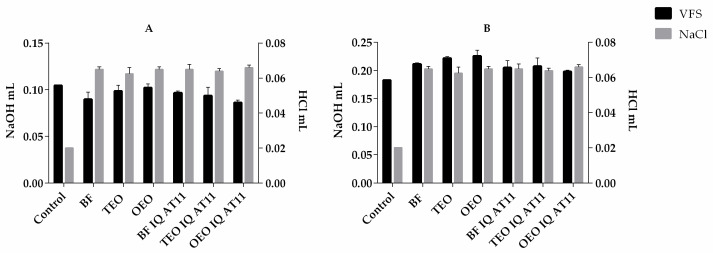
(**A**) Relevant and (**B**) absolute pH buffering capacity of formulations with dispersion in 0.9% NaCl (NaCl columns in gray) and vaginal fluid simulant (VFS columns in black). Titration was performed using HCl (1M) since all formulations had pH > 5 in the dispersion experiments performed in NaCl, and in VFS, NaOH (1M) was used since the pH changed to less than 5 until pH > 9.

The maintenance of the vaginal acidic pH (3.5–4.5) contributes to normal vaginal physiology and microbiota but can vary due to the menstrual cycle phase, hormonal stimulation, and the presence or absence of infections [32,37]. Formulations’ pH and buffering capacity are important to evaluate, as they must be compatible with the vaginal pH. To evaluate the formulation buffering capacity, we considered two different endpoints regarding the pH buffering capacities: the relevant buffering capacity (RBC) and the absolute buffering capacity (ABC). Figure 3 shows the relevant and absolute buffering capacities for all the formulations studied, either dissolved in VFS or normal saline solution (NaCl 0.9%). The pH values of the formulations were above 5 when mixed with the saline solution but within the pH range considered normal for reproductive-aged women when mixed with VFS. All formulations showed higher buffering capacities after being mixed with VFS and NaCl [38]. Compared with the control, the buffering capacity of the formulations when diluted with VFS does not have significant changes, but when mixed with NaCl, this parameter increases. These results show that after dilution with VFS, all formulations acquire the pH of this solution and do not change its properties, indicating that they are not expected to change the vaginal pH from the acidic range (although the natural pH of the gels is slightly higher than the ideal pH). The acidic pH of the vaginal environment contributes to the normal functioning of physiological processes that help to favor the microbiota, promoting a balanced immune response; hence, vaginal products should be compatible with the vaginal pH and preferably preserve it or even aid in its recovery in cases of disbalance (e.g., bacterial infections, hormonal changes). Although the formulations did not show a significant buffering capacity when diluted in VFS in comparison with the control group, this result is beneficial, considering that the naïve pH of the formulation is slightly higher than the ideal range, so it is expected that they maintain the physiological pH of the vaginal environment.

#### 3.2.2. Viscosity

Rheological profiles were determined directly and after dilution in VFS at 37 °C of the four formulations (TEO, OEO, TEO IQ AT11, and OEO IQ AT11). These dilutions can more closely resemble the rheology that vaginal products adopt after application. The objective of evaluating the viscosity of the formulations is to predict their distribution and retention in the genital cavity, considering that these two are still a challenge for semisolid formulations and greatly impact the efficacy of the products [38].

The results are presented in Figure S4 by the viscosity in mPa.s at velocities of 50 and 150 RPM (directly) and 50 and 250 RPM (dilution). TEO and OEO formulations have lower viscosity values when diluted with VFS, an expected result according to previous studies since the vaginal simulant is mainly water and salts; therefore, it has almost no viscosity [32,38]. It is also seen that the viscosity is greater at a lower velocity of 50 RPM than at 150 RPM, as expected, showing that both formulations are shear-thinning fluids. A slight increase in the viscosity was shown in both velocities when the liposomes-functionalized gel was diluted in VFS. These results are suitable since more viscous formulations are more likely to present a longer retention time in the vaginal canal and are less likely to leak, but the difference between these formulations is not relevant.

Previous studies [32] evaluated the viscosity of different commercial vaginal products Gino-Canesten^®^ and Gyno-Pevaryl^®^, and gels like Blissel^®^. Since the composition of each product determined its unique behavior, directly measured formulations cannot accurately anticipate the acquired viscosity after vaginal administration. In general, the viscosity was lower when the vaginal products were diluted in VSF, except for Blissel^®^ [32]. Another study conducted by Aka-Any-Grah et al. reports differences among formulations developed with HPMC and F127/F68, especially on rheological properties, where diluted formulations had higher viscosity values than non-diluted ones [39].

Both formulations have HPMC in their composition, a cellulose derivative polymer, which explains the high viscosities [40], but only TEO IQ AT11 and OEO IQ AT11 are functionalized with Lipo IQ AT11. This last formulation presented slight variations of viscosity (for both speeds) when in an environment that mimics the vaginal medium compared to the direct formulation, proving to be not quite resistant to dilutions in physiological fluids. Although lower viscosity allows for better dispersion of a product, these results are a positive outcome that could circumvent problems associated with leakage and low residence time in the genitourinary tract, allowing for the formulation to be more in contact with the vaginal mucosa [32].

#### 3.2.3. Osmolality

According to an advisory note published by the WHO in 2012 [41], the osmolality of a personal lubricant should not exceed 380 mOsmol/kg to minimize any risk of epithelial damage, noting that high osmolalities are correlated with their potential for vaginal irritation. However, due to many lubricants presenting higher osmolality values than this limit, the WHO increased it to 1200 mOsmol/kg since broad intervals of osmolality are well tolerated in vaginal administration [32]. Table S1 presents the osmolality results for all formulations, which showed osmolalities slightly above the upper limit recommended by the WHO. These values of osmolality are higher than those obtained for the original formula of Universal Placebo and can be related to the presence of glycols. In this case, there is propylene glycol, which is known to increase this parameter and is absent from the original formulation [42]. In fact, the WHO recommends that glycerin, another glycol used in the pharmaceutical industry, and propylene glycol should not exceed 9.9% (*w*/*w*) and 8.3% (*w*/*w*), respectively [41]. Considering this, the results agree with the literature, noting that 8% of propylene glycol was used to produce these formulations. This excipient was added to this formulation to improve the penetration of the drugs in the vaginal epithelia, promoting efficacy. Dilutions are crucial to know the product-associated ability to cause irritation in the vaginal canal and provide a better understanding of the in vivo behavior of the product. All the osmolality values decreased after the mixture with VFS were statistically different from direct formulations and were in accordance with the recommended WHO criteria. This result was expected since VFS is a hypotonic solution. Although the formulations presented higher values than those recommended by the WHO, after dilution with VFS, all conditions were within the suggested range, therefore decreasing the possibility of irritation and assuring safety for the vaginal epithelium. Also, it must be noted that these products are intended for treatment and not for chronic use.

#### 3.2.4. Bioadhesion

To conclude the characterization of the formulations, a bioadhesion assay was performed. This experiment allows for a better understanding of the product’s ability to adhere to a biological surface [32]. Bioadhesive drug delivery systems prolong the residence time of the product in the application site, facilitating contact of the dosage form with the absorption surface, leading to a better therapeutic performance of the drug [43]. Mucus is a viscous and adhesive secretion produced by glandular columnar epithelial cells with multiple purposes, including lubrication, sustaining a moist epithelial environment, acting as a protective barrier against pathogens and toxins, and facilitating the exchange of gases and nutrients through its layered structure [44]. Predominantly composed of mucins, mucus derives its heterogenous mesh-like structure from these secreted proteins [32]. Two key processes make up the adhesion mechanism. Firstly, there is a main contact stage in which hydration and spreading are the most crucial phases. Next comes a consolidation stage that strengthens the polymer–mucin junction through weak van der Waal and hydrogen bonds interactions [40]. For these reasons, the best polymers to use are those with a high number of functional groups, like hydroxyl and carboxylate groups. Table S2 represents the obtained results of all parameters. The peak force of adhesiveness corresponds to the force applied for the failure to happen, while the debonding distance is the distance to undergo the same process [37]. Finally, the work of adhesion is the work to separate the two surfaces and was the parameter taken into consideration for the one-way ANOVA statistics. This statistical test determined that the formulation OEO IQ AT11 has a statistical difference from the control (performed without any formulation). Numerous polymers known to have mucoadhesive properties are described in the recent literature [40], for example, chitosan derivatives, hyaluronic acid, and in the case of the formulations in the study, cellulose derivatives, like HPMC. Pharmaceutical forms and polymer’s ability to adhere also depend on the characteristics of the vaginal epithelial environment; therefore, this should be considered while evaluating them [40]. As seen in Table S2, OEO IQ AT11 showed a greater bioadhesion, as it was necessary for a higher detachment force. These results might be due to the presence of OEO and the nanosystem, which makes the formulation more viscous, leading to a higher detachment force needed for it to separate from the vaginal tissue. The control presented the lowest work of adhesion, as expected since it has no formulation in the vaginal tissue. Pharmaceutical forms and polymers’ ability to bioadhere also depend on the characteristics of the vaginal epithelial environment; therefore, this should be considered while evaluating them [43]. Bioadhesive dosage forms can offer prolonged in situ residence, which has benefits including fewer administrations needed, reduced vaginal leakage, and allowing for close contact between drugs and the epithelial tissue [37]. However, these results must be carefully analyzed considering the standard deviations presented for each parameter, which can result from the fact that each measurement was made in different porcine tissues.

### 3.3. In Vitro and Ex Vivo Cell Assays

#### Cell Viability Assay

**Figure 4:**
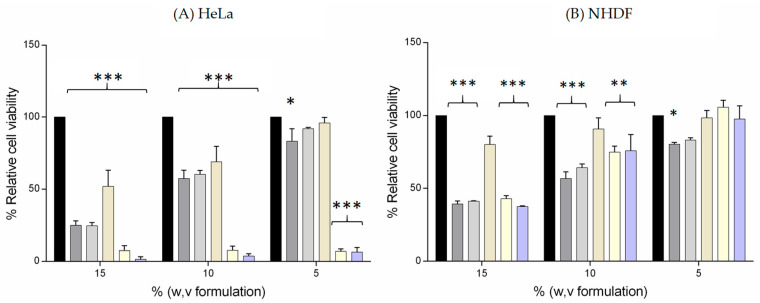

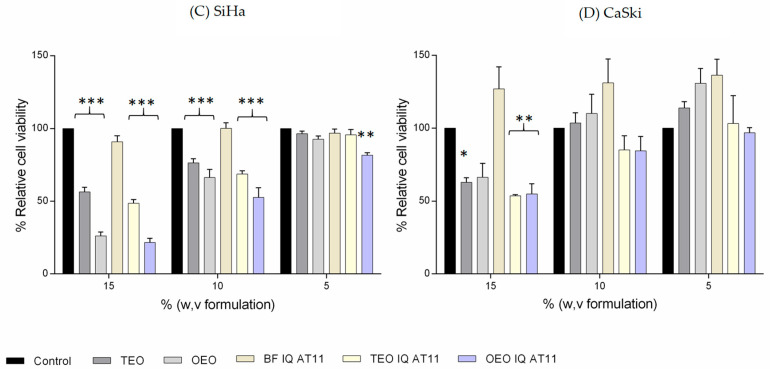
Percentage of relative cell viability of all formulations after 24 h of incubation at 37 °C in (**A**) HeLa, (**B**) NHDF, (**C**) SiHa, and (**D**) CaSki cell lines. The results correspond to the mean and standard error of three measurements. *, **, and *** represent statistically different values from the control (two-way ANOVA, * *p* < 0.05, ** *p* < 0.001, *** *p* < 0.0001, Dunnett’s multiple comparisons test).

Cell viability studies are important for testing formulations intended for vaginal application on the same type of cancerous cells found in the cervix. Therefore, three types of cancerous cell lines—HeLa (HPV18 positive), SiHa (HPV16 positive), and CaSki (HPV16 and 18 positive)—along with one healthy cell line (NHDF) were employed to assess the cytotoxicity of the formulations. Different concentrations of the formulations, comprising essential oils at a concentration of 0.25% and liposomes at a concentration of 1%, were incubated with the cells for 24 h. The obtained results are shown in Figure 4.

Most of the formulations presented a linear increase in cell viability with a decrease in the concentration in a typical dose–response relation. Overall, the formulations have a decrease in viability more pronounced in the HeLa cell line than in the SiHa and CaSki cell lines and are more evident at a concentration of 15%. SiHa cells did not show as promising results as HeLa cells but demonstrated a decrease in cell viability in concentrations of 15% and 10%. Finally, CaSki cells were shown to have smaller to no effect in concentrations of 5% and 10%; however, a 15% concentration is shown to still have an effect in these cells.

NHDF cells are normal dermal fibroblast cells and are used as a control. The relative viability was greater than 50% for all formulations in the concentrations of 10 and 5%, and it was about 40% for all formulations at a concentration of 15%. Previously, Lopes-Nunes et al. performed a study to evaluate the effect of IQ when conjugated with DNA aptamer-functionalized gold nanoparticles, and the results suggest that the cervical cancer cells have a higher sensitivity to IQ when compared to normal cells [45].

TEO and OEO, conditions that only contain the essential oil-infused base formulation, significantly decreased cell viability in all concentrations proportionally. This occurrence is related to the cytotoxic activity of the thyme and oregano essential oils, which was already reported in the literature [6,8]. Preljevic et al. performed a study to evaluate the anticancer effect of *Thymus vulgaris*, and they observed that this oil induced apoptosis in cervical adenocarcinoma HeLa cells by activating caspase-3 and caspase-8 [46]. They also observed an increase in the levels of miR-16 and miR-34a in the HeLa-treated cells, suggesting prominent anticancer properties [46]. Although these phenomena are not well described in the literature for the *Oregano vulgare*, these essential oils have demonstrated positive outcomes in HeLa (adenocarcinoma) [8,46,47], T47D (breast cancer) [48], and HCT116 (colorectal cancer) [6] cell lines, as well as inhibiting growth in leukemia THP-1 cells [6]. In a typical dose–response relationship, all the formulations demonstrated a linear rise in cell viability with decreasing concentration.

**Figure 5:**
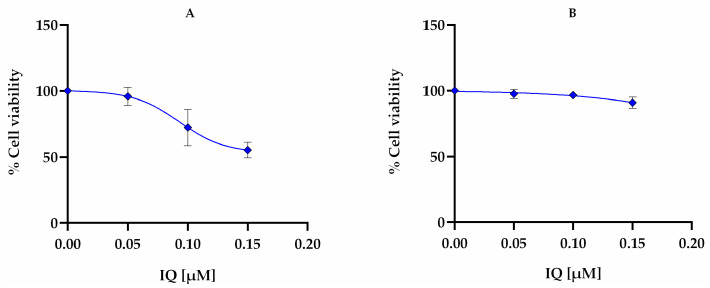
Comparative dose–response relationship of IQ (BF IQ AT11) in (**A**) HeLa and (**B**) NHDF cells after 24 h of incubation at 37 °C.

When conjugated in the base formulation with Lipo IQ AT11 (BF IQ AT11), cell viabilities were lower than 100% but were not lower than 80%, as was the case of the 15% concentration. This can be due to the functionalization of the liposomes with AT11 that specifically binds to nucleolin (with high affinity), which is overexpressed at the surface of the cervical cancer cells [45]. When comparing the effect of BF IQ AT11 on HeLa and NHDF cells (Figure 5), it is evident that the liposomes exhibit some selectivity towards cancer cells. This selectivity can be attributed to the AT11 aptamer, which has been previously shown to enhance the targeting of anticancer drugs specific to HeLa cells [22]. Finally, the formulations, TEO IQ AT11 and OEO IQ AT11, which have all the components, showed some cytotoxicity, especially in higher concentrations and, once again, due to the presence of the essential oils.

Lower antiproliferative action was observed in SiHa cells. In the lowest concentration used, none of the formulations had a significant decrease in cell viability, while in the 15% concentration, both TEO IQ AT11 and OEO IQ AT11 had cell viabilities less than 50%. As shown in Figure 4, none of the formulations at any concentration had a significant cytotoxic effect compared with the control in the CaSki cells. These results must be examined with caution since none of the conditions had statistically reliable results.

As described in the literature [49], IQ is generally used in lesions caused by HPV and, additionally, it is suggested that it also has anticancer effects on malignant cells resulting from high-risk HPV infections.

Finally, the final formulations, TEO IQ AT11 and OEO IQ AT11, showed a high cytotoxic effect in all cervical cancer lines and were more accentuated in the concentration of 15%. Due to the rise in multidrug resistance and the unfavorable side effects of conventional chemotherapeutic agents, several researchers examined the possible therapeutic benefits of plant essential oils in anticancer treatment [8]. It is plausible that this effect is due to the presence of the essential oil since BF IQ AT11 at the same concentration had no significant changes. However, the formulations with the essential oils showed lower cell viability than the final formulations, which suggests a possible synergistic effect of the oils with the nanosystem.

In summary, the results suggest that the synergistic action of combining the essential oils and the liposomes with the anticancer drug Lipo IQ AT11 resulted in a decrease in the growth of all the cervical cancer cell lines compared to NHDF cells.

### 3.4. Confocal Fluorescence Microscopy

The cellular uptake of Lipo IQ Cy5-labelled AT11 was studied in NHDF, HeLa, SiHa, and CaSki cells by confocal microscopy. AT11 was conjugated with Cy5 to follow its fluorescence and to visualize its cellular uptake.

**Figure 6:**
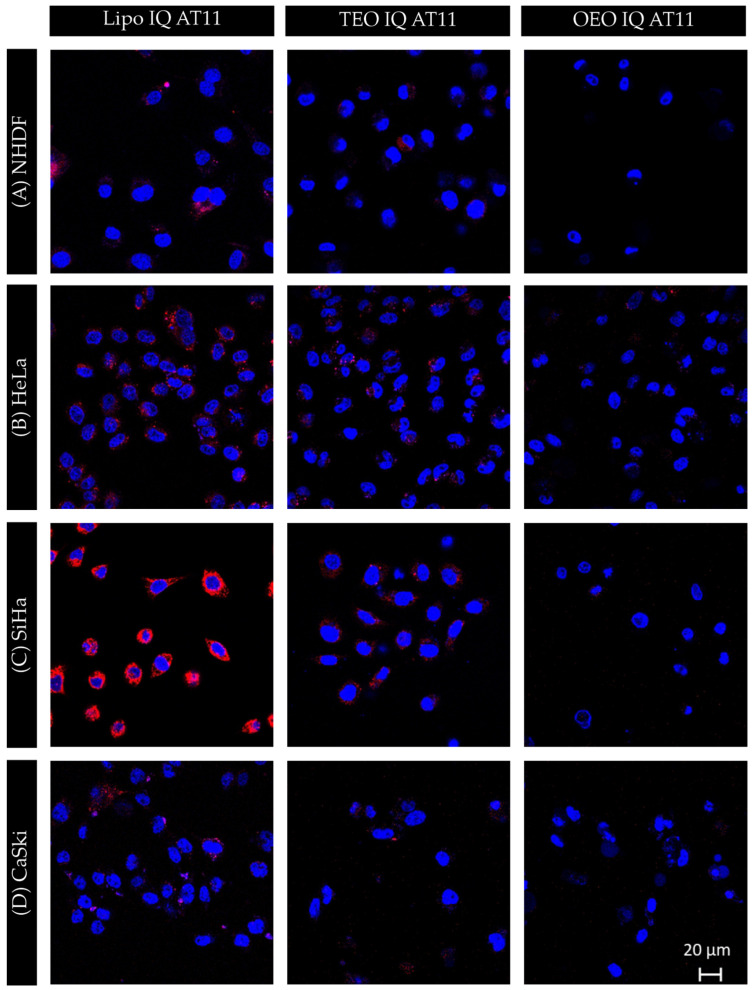
Confocal fluorescence images of (**A**) NHDF, (**B**) HeLa, (**C**) SiHa, and (**D**) CaSki cell lines incubated with IQ liposomes (Lipo IQ AT11), thyme formulation with liposome IQ (TEO IQ AT11), and oregano formulation with liposome IQ (OEO IQ AT11) for 24 h at 37 °C. Cell nuclei are stained with Hoechst 33342 (blue), and Cy5-AT11 is shown in red.

In Figure 6, the images obtained by confocal microscopy are presented. NHDF cells internalized more Lipo IQ AT11 than expected. This may be due to the presence of IQ, allied to the fact that normal cells can also internalize AS1411, a very similar aptamer to AT11 [50]. Figueiredo et al. [22] carried out a study in which they observed that even in the absence of nucleolin overexpressed on the cell surface, NHDF cells were able to internalize AT11 [22]. It was proposed that endocytosis was the responsible mechanism for this initial uptake, assuming that the aptamer is gradually cleared from normal cells by efflux or exocytosis [22], which could explain the liposomes internalization by NHDF cells and the absence of effects in the cell viability [51], as observed by the MTT assay (Figure 4). However, this cell line still presented less internalization of the final formulations (TEO IQ AT11 and OEO IQ AT11) in comparison to all cancer cell lines. HeLa cells show insignificant internalization of the formulations with the essential oils but showed higher intensity of Lipo IQ AT11 in the following order: Lipo IQ AT11, TEO IQ AT11, and OEO IQ AT11. SiHa cells presented a higher internalization of all the conditions tested than the other cell lines, especially in the condition Lipo IQ AT11. OEO IQ AT11 had the lowest internalization in all cell lines when compared with the formulation with TEO, which suggests that even though it internalizes less into the cells, it can have significant effects on reducing cell viability (Figure 4), especially in cancer cells.

In general, after incubation with Lipo IQ AT11, the formulations were internalized by the cells and localized in the cytoplasm compartment, as described previously [45]. The fact that the formulations are internalized by the cells is clear, according to the results presented in Figure 6.

### 3.5. Permeation of the Formulations in Vaginal Tissue (Ex Vivo Studies) and Validation of the HPLC–FLD Method

Evaluating the permeation of drugs is an important aspect of pharmaceutical development, especially when formulating drugs for targeted delivery to specific areas, like the vaginal wall. The selection of an appropriate testing method is critical to accurately replicate the in vivo environment. We used porcine ex vivo tissue, the most frequently animal model used due to the physiological and morphological similarities, easy handling, and low costs associated [52]. The permeation of IQ was evaluated in the Ussing chamber, and the results were quantified by HPLC. The formulations without the active compound in their composition (TEO and OEO) were used as the controls. The HPLC quantification was performed for the vaginal tissue, donor, and receiving chamber for the samples collected at the following times: 0 min, 5 min, 15 min, 30 min, 1 h, 2 h, 3 h, 4 h, 5 h, 6 h, 12 h, 24 h, 48 h, and 72 h. Based on the outcomes obtained from the HPLC analysis, the permeation and persistence of IQ in the analyzed aliquots display a seemingly erratic pattern. IQ was detectable at specific time points in each withdrawn aliquot, indicating a high degree of permeation with a somewhat unpredictable pharmacokinetic profile associated with the release and accumulation of this compound in the vaginal epithelial tissue. It is noteworthy that IQ showed a high susceptibility to degradation when exposed to direct sunlight, although a dedicated stability study was not conducted. In contrast, the examination of tissue samples indicates a notable concentration and accumulation of the IQ compound. This finding suggests that IQ is retained within the tissue, supporting its potential for topical application to enhance bioadhesion to the vaginal epithelium while also considering the permeation results. Further testing is warranted to establish the optimal equilibrium between the amount of active ingredients permeating the vaginal tissue and the amount retained in the tissue. This balance is a crucial factor for ensuring both safety and efficacy in vivo.

HPLC-FLD method validation followed the guidelines outlined by the Food and Drug Administration (FDA) for the analysis of drugs and biologics (https://www.fda.gov/regulatory-information/search-fda-guidance-documents/analytical-procedures-and-methods-validation-drugs-and-biologics accessed on the 8 January 2024). Nine calibrators (*n* = 5) were established within the linearity range between 0.0039 and 1 mM, and additionally, four quality controls (0.0039, 0.016, 0.25, and 1 mM) (*n* = 3) were included. The criteria used included a weighted determination coefficient (R^2^) higher than 0.99, and the accuracy of the calibrators within ±15% from the nominal value (except at the low limit of quantitation, LLOQ, where ±20% was accepted) was adopted as acceptance criteria. The adopted calibration range was wide, and as such weighted least squares regression had to be used to compensate for heteroscedasticity (1/x). Table S3 shows the calibration obtained data. The LLOQ was calculated based on the calibration curve (and not using the signal-to-noise approach), for which a criterion of a coefficient of variation (CV) ≤ 20% and a mean relative error (RE) of ±20% was established. This value was 0.0039mM. According to FDA guidelines, the limit of detection (LOD) is defined as the concentration that yields a signal-to-noise ratio > 3. In this work, the limit of detection (LOD) was not systematically studied and the same concentration value for the lower limit of quantification (LLOQ) was assumed due to the signal of the LLOQ being higher than 3.

Precision and accuracy were evaluated during the 5-day protocol, adopting the same concentrations used for the quality controls. CVs equal to or lower than 15% were accepted for precision at all studied concentration levels, while for accuracy, an RE of ±15% (from the nominal concentration) was accepted for all concentrations, except the LLOQ (±20%).

The CVs obtained in the study of intra-day precision and accuracy (RE) were typically lower than 4%, with an accuracy ranging from 97 to 112%. Inter-day and accuracy were evaluated on the same day by the analysis of five replicates at 0.04 (LLOQ), 1.25, 10, and 40 μg/mL. The obtained CVs were once again within the accepted criteria, with the CVs lower than ±10%, and the accuracy ranged from 95 to 111%.

### 3.6. Microbiology Assay

#### Minimal Inhibitory Concentration

The minimal inhibitory concentration using the microdilution method is only possible if the negative controls are clear and the positive controls show turbidity [53]. TEO and OEO are at a concentration of 1% in the formulation and the liposomes are at a concentration of 1%. The microorganisms studied were *Candida albicans*, *Aspergillus brasiliensis*, and *Staphylococcus aureus*. The choice of the strains was made based on the local application of the gel and the possible infections that may occur, and the visual MIC and MIC_50_ results are shown in Table S4. Both BL and BL AT11 did not have effects in the studied strains; however, when combined with thyme essential oil, BL AT11 was shown to inhibit growth at a 10% concentration for *Candida albicans*, 2.5% for *Aspergillus brasiliensis,* and 5% for *Staphylococcus aureus*. When combined with OEO, BL AT11 liposomes were only shown to inhibit growth at a 2.5% concentration for *Aspergillus brasiliensis* and 10% for *Staphylococcus aureus*.

TEO IQ AT11 also inhibited microorganism growth at a 5% concentration for *Candida albicans*, 5% for *Aspergillus brasiliensis,* and 10% for *Staphylococcus aureus*. These results refer to the visual MIC, which means the last blue well in the column of the desired condition. BL and BL AT11 formulations did not seem to have a typical dose–response pattern, and almost no inhibition was seen in these conditions. However, formulations with TEO showed a typical curve where the viability decreases with the increase in concentration for *Aspergillus brasiliensis* and *Candida albicans*. The same was not seen in *Staphylococcus aureus*, where a slight increase in viability was seen in 0.625% and 1.25% concentrations; however, the results do not seem to have statistical differences.

TEO and OEO have high concentrations of thymol and carvacrol, which contribute to their antifungal properties [8]. Their antifungal effect is linked to the disruption of the fungal cell wall integrity and the interference with the formation of ergosterol [8]. The oils’ phenolic alcohol content directly affects the antifungal activity, confirming the widely held belief that their antimicrobial efficacy is determined by their chemical composition [54,55]. Borugă et al. [54] evaluated the antimicrobial activity of thyme in various microorganisms, including *Staphylococcus aureus* and *Candida albicans*. The results demonstrated the effectiveness of thyme and concluded that antimicrobial activity is a synergism between all components within the essential oil. To evaluate the antifungal properties of OEO and its main components (carvacrol and thymol), Manohar et al. [56] performed a study against *Candida albicans* both in vitro and in vivo and also examined the efficacy of amphotericin B and nystatin. The results showed that OEO has the potential to act as an antifungal, amphotericin B, and nystatin [56]. So, the decreased cell growth might be due to the presence of the essential oils and their compounds, thymol and carvacrol, since there was no growth inhibition in the conditions without the oils (BL and BL AT11).

Briefly, all the results above demonstrated that the formulation is toxic for fungus *Candida albicans*, *Aspergillus brasiliensis*, and bacteria *Staphylococcus aureus* in the presence of TEO and OEO at 1% in the formulation, and the effect of Lipo IQ and Lipo IQ AT11 is not very pronounced.

Given the results obtained from the physiological, biological, and microbiological characterization, it would also be important to assess the pharmacokinetics and biodistribution of the nanosystems to predict their efficacy and safety in clinical environments, proceeding to the in vivo experiments. A relevant model for assessing the safety and pharmacokinetics of these formulations would be K14-HPV16, transgenic mice, which are characterized by the development of a variety of histological lesions in the skin of the region, from early hyperplastic to invasive squamous cell carcinomas, and are capable of mimicking those seen in the cervical region of women [57,58].

## 4. Conclusions

In this study, Lipo IQ AT11 was produced and subsequently incorporated into vaginal formulations containing thyme or oregano for the treatment of intraepithelial lesions caused by HPV. To achieve this, lipidic nanoparticles were produced using the ethanol injection method. The size, zeta potential, and drug release characteristics of these nanoparticles were assessed. The final sizes ranged between 110 and 160 nm, with a polydispersity index (PDI) below 0.3, demonstrating the suitability of this method for liposome production. The successful functionalization of AT11 was confirmed through acrylamide gel analysis. Drug loading and release assays revealed that IQ was effectively associated with the membrane and could be released from the nanosystem during the first 24 h. Technological properties refer to attributes and characteristics that significantly impact the performance, stability, and suitability of formulations for vaginal administration, playing a crucial role in ensuring the safety and effectiveness of vaginal products. The produced liposomes were incorporated into formulations containing essential oils (TEO or OEO), and subsequently, they were technologically characterized. Buffering capacity experiments provided evidence suggesting that the formulations are likely to maintain the physiological pH of the vagina after administration. Relative to the viscosity, the formulations exhibited elevated levels, ensuring their suitability for mitigating discomfort and preventing leakage, while still facilitating gel dispersion and contact with the vaginal environment. The assessment of osmolality is crucial to prevent possible irritation issues. The formulations were found to be within the recommended range for vaginal lubricants, especially after dilution with VFS, thereby guaranteeing the safety of the product for administration. Finally, the evaluation of bioadhesion allowed for an understanding of the adhesive behavior between the gel and the vaginal tissue, and the final formulations demonstrated promising results. When tested in vitro, the formulations demonstrated the ability to significantly reduce cell viability, particularly in cervical cancer HeLa cells. However, the cell viability results for CaSki and SiHa cells were unexpectedly higher, warranting further studies to better understand the mechanism of action. This notable effect was primarily attributed to the presence of essential oils, as the impact was considerably lower in their absence. It is noteworthy that a reduction was also observed in NHDF cells. Therefore, the optimal concentration of essential oils must be carefully selected to balance efficacy while minimizing potential effects on normal cells. Furthermore, the produced liposomes contributed to an enhanced selectivity of IQ towards HeLa cells compared to NHDF cells, as demonstrated by the BF IQ AT11 at the 15% and 10% concentrations. Additionally, confocal microscopy revealed an accentuated accumulation of liposomes within cervical cancer cells, emphasizing their potential for targeted delivery. Concerning the microbiology assays, the results showed that the formulations have antimicrobial and antifungal properties against the microorganisms studied.

In summary, although the outcomes are promising for the advancement of nanosystems as potential treatments for HPV-induced lesions, addressing challenges associated with translating preclinical research to clinical applications is essential. The liposome-based delivery systems for imiquimod offer significant advantages in terms of biocompatibility, targeted delivery, and controlled release, making them a promising option for treating HPV-induced lesions. However, advancements in stability enhancement, for developing more stable liposome formulations to prevent leakage and degradation, are essential for their widespread application. Also, conducting in vivo efficacy studies in relevant animal models is necessary to understand the pharmacokinetics and biodistribution of nanosystems and to predict their efficacy and safety in clinical environments.

## Figures and Tables

**Table 1:** Average size and PDI of blank liposomes (BLs), blank liposome + AT11 (BL AT11), liposomes with IQ (Lipo IQ), and liposomes with IQ + AT11 (Lipo IQ AT11).

	Z-Ave (nm (±SD))		PDI (±SD)	
	Day 0	Day 30	Day 0	Day 30
BL	129.6 ± 1.3	130.9 ± 0.4	0.135 ± 0.030	0.094 ± 0.020
BL AT11	160.2 ± 2.5	113.27 ± 0.7	0.225 ± 0.010	0.135 ± 0.020
Lipo IQ	111.4 ± 1.0	116.9 ± 0.9	0.135 ± 0.030	0.153 ± 0.020
Lipo IQ AT11	117.4 ± 0.7	113.3 ± 0.7	0.225 ± 0.030	0.125 ± 0.020

## Data Availability

Data is contained within the article and Supplementary Materials.

## Supplementary Materials

The following supporting information can be downloaded at: https://www.mdpi.com/article/10.3390/pharmaceutics16070864/s1. Figure S1: Synthesis of Lipo IQ AT11 and gel production with 0.25% of thyme or oregano essential oils and Lipo IQ AT11; Figure S2: Particle size distribution diagram for the liposomes BL, BL AT11, Lipo IQ and Lipo IQ AT11 on the left side, on the day they were prepared and on the right side, after 30 days; Figure S3: Acrylamide gel of (1) 25bp Marker, (2) AT11-TEG-Cholesteryl, (3) Lipo IQ (1mg) + AT11-TEG-Cholesteryl (1 nmol), (4) Lipo IQ (1mg) + AT11-TEG-Cholesteryl (2 nmol); Figure S4: Viscosity comparisons for direct and diluted measurements at 37 °C. The dilutions were made in VFS. The results are presented as mPa.s, corresponding to the mean and standard deviation of three measurements (*n* = 3); Table S1: Osmolality results of the formulations studied, *n* = 3. BF–Base Formulation. TEO—Base Formulation with 0.25% Thyme. OEO—Base Formulation with 0.25% Oregano TEO IQ AT11—Base Formulation with 0.25% Thyme and 1% of Lipo IQ AT11 and OEO IQ AT11—Base Formulation with 0.25% Oregano and 1% of Lipo IQ AT11. **** Represents statistically different from BF used as control, two-way ANOVA, *p* < 0.0001; Table S2: Bioadhesive parameters (Peak force adhesiveness (N), debonding distance (mm), and work of adhesion (N.mm)) determined for the base formulation (BF), Base Formulation with 0.25% Thyme and 1% Lipo IQ AT11 (TEO IQ AT11) and Base Formulation with 0.25% Oregano and 1% Lipo IQ AT11 (OEO IQ AT11), *n* = 4. Vaginal tissue without formulation was used as control.* Represents statistically different from control two-way ANOVA, *p* < 0.05; Table S3: Linear range, calibration curve, correlation coefficients, and LLOQ (*n* = 5); Table S4: Results of the visual MIC and MIC 50% for all the formulations in *Candida albicans*. BL-blank liposome, BL AT11—blank liposomes with AT11, TEO AT11—base formulation with the thyme essential oil and BL AT11, OEO AT11—base formulation with the oregano essential oil and BL AT11, TEO IQ AT11—base formulation with the thyme essential oil and Lipo IQ AT11 and OEO IQ AT11—base formulation with the oregano essential oil and Lipo IQ AT11.

## Abbreviations

CIN	Cervical intraepithelial neoplasia
DLS	Dynamic Light Scattering
FDA	Food and Drug Administration
HPMC	Hydroxypropyl methylcellulose
HPV	Human papillomavirus
IQ	Imiquimod
MHB	Mueller Hinton Broth
MIC	Minimal inhibitory concentration
NCL	Nucleolin
PDA	Potato dextrose agar
PDI	Polydispersity index
SDA	Sabouraud dextrose agar
TSA	Tryptone Soy Agar
VFS	Vaginal fluid simulant
VIN	Vulvar intraepithelial neoplasia
WHO	World Health Organization
