# Supplementary material for: Imiquimod-Loaded Nanosystem for Treatment Human Papillomavirus-Induced Lesions
Source: Pharmaceutics. 2024 Jun 27;16(7):864. doi: 10.3390/pharmaceutics16070864 (PMC11279553; doi:10.3390/pharmaceutics16070864)
Supplement: Supplementary file 1 [file pharmaceutics-16-00864-s001.zip › pharmaceutics-3016637-supplementary.pdf]

Supporting Information

Imiquimod-Loaded Nanosystem for Treatment Human Papillomavirus-Induced Lesions

Izamara Gomes Maocha[1†], Beatriz Rosado[1†], Jéssica Lopes-Nunes[1], Melanie Lopes[1], Joana Rolo,[1] Bruno Pires[1], Eugénia Gallardo,[1] Ana Palmeira-de-Oliveira,[1,2,3] José Martinez-de-Oliveira,[1] Rita Palmeira-de-Oliveira,[1,2,3] Rui Medeiros[5,6], Carla Cruz[1,4*]

[1]CICS-UBI - Health Sciences Research Centre, University of Beira Interior, Covilhã, Portugal;

[2] Labfit–Health Products Research and Development Lda, UBImedical, Estrada Nacional 506, 6200-284, Covilhã, Portugal;

[3] Faculty of Health Sciences, University of Beira Interior, Av. Infante D. Henrique, 6200-506, Covilhã, Portugal;

[4] Departamento de Química, Universidade da Beira Interior, Rua Marquês de Ávila e Bolama, 6201-001, Covilhã, Portugal.

[5] Molecular Oncology and Viral Pathology Group, Research Center of IPO Porto (CI-IPOP)/RISE@CI-IPOP (Health Research Network), Portuguese Oncology Institute of Porto (IPO Porto)/Porto Comprehensive Cancer Center (Porto.CCC) Raquel Seruca, Porto, Portugal;

[6] Faculty of Medicine, University of Porto (FMUP), Porto, Portugal.

† These authors contributed equally to this work

*Corresponding author: carlacruz@fcsaude.ubi.pt

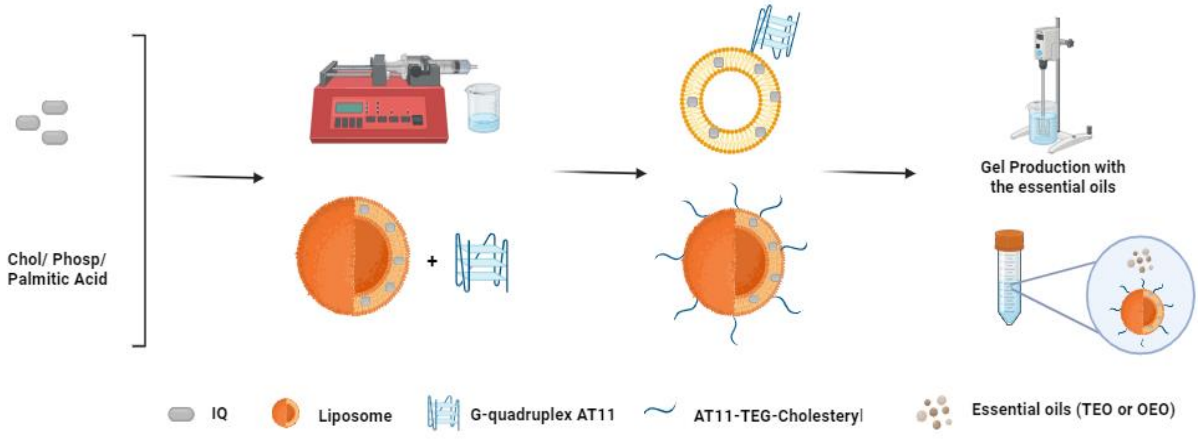

Figure S1: Synthesis of Lipo IQ AT11 and gel production with 0.25% of thyme or oregano essential oils and Lipo IQ AT11.

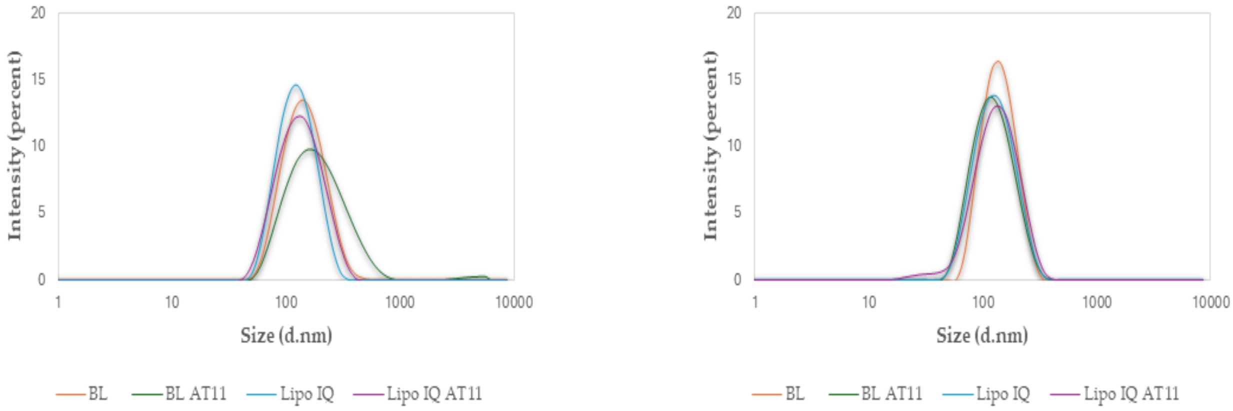

Figure S2 - Particle size distribution diagram for the liposomes BL, BL AT11, Lipo IQ and Lipo IQ AT11 on the left side, on the day they were prepared and on the right side, after 30 days.

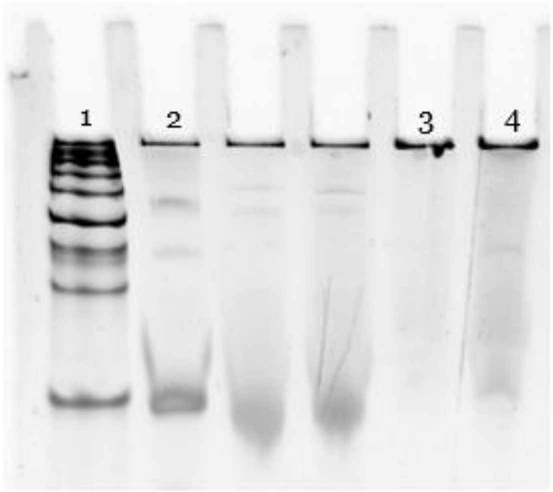

Figure S3 - Acrylamide gel of (1) 25bp Marker, (2) AT11-TEG-Cholesteryl, (3) Lipo IQ (1mg) + AT11-TEG-Cholesteryl (1 nmol), (4) Lipo IQ (1mg) + AT11-TEG-Cholesteryl (2 nmol).

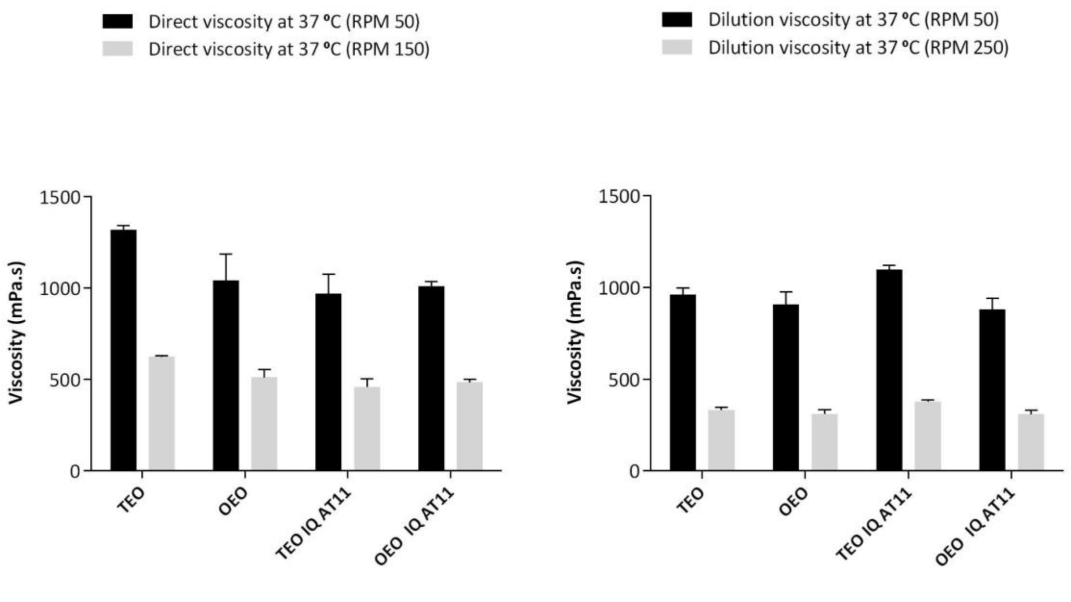

Figure S4 - Viscosity comparisons for direct and diluted measurements at 37 °C. The dilutions were made in VFS. The results are presented as mPa.s, corresponding to the mean and standard deviation of three measurements (n = 3).

Table S1: Osmolality results of the formulations studied, n = 3. BF – Base Formulation. TEO - Base Formulation with 0.25% Thyme. OEO - Base Formulation with 0.25% Oregano TEO IQ AT11 – Base Formulation with 0.25% Thyme and 1% of Lipo IQ AT11 and OEO IQ AT11 – Base Formulation with 0.25% Oregano and 1% of Lipo IQ AT11.**** Represents statistically different from BF used as control, two-way ANOVA, $p < 0.0001$.

	**Osmolality ± SD value (mOsmol/kg)**	
	**Direct Formulation**	**Diluted Formulation**
**BF**	1562 ± 47.6	838.0 ± 10.2****
**TEO**	1332 ± 39.8	1186 ± 22.7****
**OEO**	1366 ± 8.185	1184 ± 6.658****
**TEO IQ AT11**	1306 ± 54.2	1139 ± 28.3****
**OEO IQ AT11**	1367 ± 7.211	1165 ± 13.614****

Table S2: Bioadhesive parameters (Peak force adhesiveness (N), debonding distance (mm), and work of adhesion (N.mm)) determined for the base formulation (BF), Base Formulation with 0.25% Thyme and 1% Lipo IQ AT11 (TEO IQ AT11) and Base Formulation with 0.25% Oregano and 1% Lipo IQ AT11 (OEO IQ AT11), n = 4. Vaginal tissue without formulation was used as control.*Represents statistically different from control two-way ANOVA, $p < 0.05$.

	**Peak Force Adhesiveness (N)**	**Debonding Distance (mm)**	**Work of Adhesion (N.mm)**
**Control**	0.115 ± 0.149	0.443 ± 0.209	0.032 ± 0.035
**BF**	0.060 ± 0.017	2.332 ± 0.990*	0.070 ± 0.045
**TEO IQ AT11**	0.130 ± 0.060	1.160 ± 0.626	0.064 ± 0.040
**OEO IQ AT11**	0.338 ± 0.309*	0.608 ± 0.302	0.125 ± 0.111

Table S3- Linear range, calibration curve, correlation coefficients, and LLOQ (n=5).

Analyte	Weight	Linear range (mM)	Linearity*		R^2*	LLOQ (ng/mL)
			Slope	Intercept		
IQ	1/x	0.0039-1	342.35 ± 34.35	1.108 ± 0.1776	0.998 ± 0.004	0.0039

*Mean values ± standard deviation

Table S4- Results of the visual MIC and MIC 50% for all the formulations in *Candida albicans*. BL- blank liposome, BL AT11- blank liposomes with AT11, TEO AT11 - base formulation with the thyme essential oil and BL AT11, OEO AT11 - base formulation with the oregano essential oil and BL AT11, TEO IQ AT11 - base formulation with the thyme essential oil and Lipo IQ AT11 and OEO IQ AT11- base formulation with the oregano essential oil and Lipo IQ AT11.

Concentration (mM)	Intra-day precision		Inter-day precision	
	CV (%)	RE	CV (%)	RE
0.0039	2.99	112.28	10.14	111.00
0.016	3.27	98.71	5.28	103.71
0.25	2.55	97.55	3.93	99.95
1	4.37	97.02	8.08	95.02

	MIC_{visual} (% v/v)			MIC_{50} (% v/v)		
	**C.A**	**A.B**	**S.A**	**C.A**	**A.B**	**S.A**
**BL**	N.O.	N.O.	N.O.	N.O.	N.O.	N.O.
**BL AT11**	N.O.	N.O.	N.O.	N.O.	N.O.	N.O.
**TEO AT11**	10%	2.5%	5%	5%	2.5%	5%
**OEO AT11**	N.O.	2.5	10%	5%	0.625%	5%
**TEO IQ AT11**	5%	5%	10%	5%	5%	10%
**OEO IQ AT11**	5%	2.5%	5%	5%	2.5%	10%

N.O. – not observed
